# Grass Carp (*Ctenopharyngodon idella*) KAT8 Inhibits IFN 1 Response Through Acetylating IRF3/IRF7

**DOI:** 10.3389/fimmu.2021.808159

**Published:** 2022-01-03

**Authors:** Meifeng Li, Jihuan Hu, Huiling Mao, Dongming Li, Zeyin Jiang, Zhichao Sun, Tingting Yu, Chengyu Hu, Xiaowen Xu

**Affiliations:** ^1^ School of Life Science, Nanchang University, Nanchang, China; ^2^ Fuzhou Medical College, Nanchang University, Fuzhou, China; ^3^ State Key Laboratory of Food Science and Technology, Nanchang University, Nanchang, China

**Keywords:** IRF3 and IRF7, fish, KAT8, IFN 1 signaling pathway, acetylation

## Abstract

Post-translational modifications (PTMs), such as phosphorylation and ubiquitination, *etc.*, have been reported to modulate the activities of IRF3 and IRF7. In this study, we found an acetyltransferase KAT8 in grass carp (*Ci*KAT8, MW286472) that acetylated IRF3/IRF7 and then resulted in inhibition of IFN 1 response. *Ci*KAT8 expression was up-regulated in the cells under poly I:C, B-DNA or Z-DNA stimulation as well as GCRV(strain 873) or SVCV infection. The acetyltransferase domain (MYST domain) of KAT8 promoted the acetylation of IRF3 and IRF7 through the direct interaction with them. So, the domain is essential for KAT8 function. Expectedly, KAT8 without MYST domain (KAT8-△264-487) was granularly aggregated in the nucleus and failed to down-regulate IFN 1 expression. Subcellular localization analysis showed that KAT8 protein was evenly distributed in the nucleus. In addition, we found that KAT8 inhibited the recruitment of IRF3 and IRF7 to ISRE response element. Taken together, our findings revealed that grass carp KAT8 blocked the activities of IRF3 and IRF7 by acetylating them, resulting in a low affinity interaction of ISRE response element with IRF3 and IRF7, and then inhibiting nucleic acids-induced innate immune response.

## Highlights

1. KAT8, serves as an inhibitor for IFN 1 response.
2. KAT8 blocks IRF3/IRF7-induced IFN 1 response.
3. KAT8 directly interacts with IRF3/IRF7 and promotes their acetylation through MYST domain.
4. KAT8 inhibits the interaction between IRF3/IRF7 and ISRE within target gene.

## Introduction

Innate immunity is the early line of defense against microbial infection in cell. It recognizes and eliminates pathogenic molecules through its pattern recognition receptors (1, 2). However, the inactivation of immune factors is crucial for maintaining host homeostasis after signal transduction. Therefore, several negative regulators are required in innate immune response.

The post-translational modifications (PTMs) of innate immune signaling molecules affect their activities in signal transduction, which are critical in regulating the expression of type I interferon (3–5). Currently, typical PTMs, including phosphorylation and ubiquitination, have been extensively studied (6–11). For instance, Trim26 promotes IRF3 ubiquitination and let its activation be cut down in the nucleus (12). RNF5 negatively regulates the antiviral response by mediating the ubiquitination and degradation of MITA (13). Other PTMs, such as acetylation and SUMOylation, are also found to be related to innate immunity and inflammation (14–16). RIG-I and MDA5 are SUMOylated by tripartite motif-containing protein 38 (TRIM38) in uninfected or early infected cells to ensure their optimal activation (17).

Acetylation is involved in many key cellular processes, including gene transcription, signal transduction, DNA damage repair, cell apoptosis, metabolism, and so on (18). Acetylaggtion of lysine residue is regulated by acetyltransferases and deacetylases. Protein acetylation regulates protein-protein interaction, protein stability, nucleic acid binding and enzymatic activity (19–21).

The MYST family of histone acetyltransferases consists of the following five members (i.e. KAT5, KAT6A, KAT6B, KAT7 and KAT8). They all have highly conserved lysine acetyltransferase domain (22, 23). They are vital components of chromatin regulatory proteins which can catalyze the reversible N^ϵ^-acetylation of histones and other non-histone proteins, and generate dynamic N^ϵ^-acetylation based on cell signals (24, 25).

Lysine (K) acetyltransferase 8 (KAT8), also termed as MOF, MYST1, MOZ, YBF2, SAS2 or TIP60 1, can acetylate histone H4 lysine 16 (H4K16) in mammalian cells (26). KAT8 consists of the N-terminal chromatin domain (for combining non-coding RNA and DNA), the zinc finger motif (for identifying specific DNA sequences), and the highly conserved C-terminal MYST histone acetyltransferase domain (for acetylating histones and non-histone proteins) (27–29). KAT8 is involved in a variety of biological processes. For instance, KAT8 acetylates NRF2 to modulate anti-drug response in human non-small cell lung cancer (30); KAT8 acetylates A1B1 to promote breast cancer cell proliferation (31); It was also found that KAT8 can catalyze acetylation of the DNA binding domain of p53 in H1299 cells to induce cell apoptosis (29). In short, KAT8 participates in DNA repair, cell apoptosis, cell proliferation, cellular lifespan, autophagy, tumorigenesis, *etc.* (31–34).

There are still a few studies on the involvement of KAT8 in innate immune response. KAT8 acetylates IRF3 to negatively regulate antiviral immunity in mice (35). However, the process of IRF3 and IRF7 acetylation remains enigmatic. Therefore, revealing the mechanism by which KAT8 acetylates IRF3 and IRF7 is vital for innate immune response.

In this study, the experimental evidences provided a new insight into how grass carp acetyltransferase KAT8 functions in the IFN 1 pathway. We found grass carp KAT8 interacted with IRF3 and IRF7 and then acetylated them, resulting in a weak interaction between IRF3/IRF7 and ISRE within target gene, finally suppressing IFN 1 expression.

## Materials and Methods

### Cell Culture, Virus Infection, Reagents, Antibodies

Grass carp (*C. idella*) ovary cells (CO), Grass carp kidney cells (CIK) and epithelioma papulosum cyprini (EPC) cells were maintained in medium 199 supplemented with 10% FCS and 1% penicillin/streptomycin solution. CIK cells are usually used in subcellular localization assay and gene knockout experiment because of their stronger adhesion and better cell morphology. CO cells are usually used in overexpression experiments due to their higher transfection efficiency.

GCRV (strain 873) and SVCV were gifts from Professor Wuhan Xiao (Institute of Hydrobiology, Chinese Academy of Sciences, Wuhan, China). GCRV-873, a strain of GCRV virus (dsRNA virus), was propagated in CIK cells at 28°C SVCV, a ssRNA virus, was propagated in EPC cells at 28°C. M199 medium was collected and stored at 80°C until use. Viral titers were determined by a 50% tissue culture-infective dose (TCID_50_) assay on CIK or EPC cells. For viral infection, CIK cells were plated for 12 h in advance and then infected with GCRV-873 or SVCV at 10^-8^ TCID_50_.

Poly(I:C) was purchased from Sigma-Aldrich (USA). B-DNA and Z-DNA were synthesized by Sangon Biotech (Shanghai, China). The sequences are presented in **Table 1**.

**Table 1:** Sequences and applications of primers used in this study.

Primer name	Primer sequence (5’-3’)	Application
KAT8-ORF-F	ATGAACAATTCATATGAGAACTGC	cDNA cloning, Prokaryotic/eukaryotic expression vector construction
KAT8-ORF-R	TCATTTTTTGGAGAACTTGGCC	
KAT8-(▲1-151 aa)-F	GAGGAAGGTGGTGGGGGTGA	
KAT8-(▲1-151 aa)-R	TCATTTTTTGGAGAACTTGGCCTG	
KAT8-(▲151-264 aa)-F1	ATGAACAATTCATATGAGAACTGC	
KAT8-(▲151-264 aa)-R1	CACGCTTTTTCTTACTGCATCT	
KAT8-(▲151-264 aa)-F1	GCCTCCAGGCAAAGAGATTTATC	
KAT8-(▲151-264 aa)-R2	TCATTTTTTGGAGAACTTGGCC	
KAT8-(▲264-487 aa)-F	ATGAACAATTCATATGAGAACTGC	
KAT8-(▲264-487 aa)-R	TCACTGACGCCACTGACACT	
IRF3-ORF-F	ATGACCCATCCAAAACCGCT	
IRF3-ORF-R	TCACTTGGTGTCACACAACTC	
IRF7-ORF-F	ATGGCAGCGATGCAGAGCAC	
IRF7-ORF-R	TTAGTCCATTGAAGGCAGAC	
KAT8-RT-F	TGGAGCCGTTCATCTTCTACAT	Real-time PCR
KAT8-RT-R	GTACCCACGACCAGTAACTCCT	
IFN1-RT-F	GTCAATGCTCTGCTTGCGAAT	
IFN1-RT-R	CAAGAAACTTCACCTGGTCCT	
ISG15-RT-F	GGTGAAAGTTGATGCCACAGTTG	
ISG15-RT-R	TTGGAAAGGGGGGTTCGTG	
vp5-RT-F	ATCGCTTCGCTGTTTATGC	
vp5-RT-R	AAGGATGCTTGGACGCTAC	
vp6-RT-F	ATGGCACAGCGTCAGTTTT	
Vp6-RT-R	TAGTCCAAGGGCGAGCGTA	
Vp7-RT-F	TGTTCCCGTCAGCCAAATG	
Vp7-RT-F	GGCAGCGAGTCAGCACCTT	
IFN-pro-F	TGGTTGGTTTTAAAGTAGGCCTAATTG	Construction of promoter vector
IFN-pro-R	CGTTTCCAAACTAGAAGAGATGCG	
ISG15-pro-F	TGGTGAATGTACTTGGCCAA	
ISG15-pro-R	GCATTAGAACTACTACAACACCAAC	
B-DNA	CTGATACTACATTGAATTCTATATATATATATATATATAGAATTCAATGTAGTATCAGA	DNA analog
Z-DNA	CTGATACTACATTGAATTCGCGCGCGCGCGCGCGCGCGCGAATTCAATGTAGTATCAGA	
ISRE-forward	GAAACTGAAACTGAAACTGAAACTGAAACTGAAACTGAAACTGAAACTGAAACTGAAACT	DNA pull-down assays
ISRE-reverse	TCAAAGTCAAAGTCAAAGTCAAAGTCAAAGTCAAAGTCAAAGTCAAAGTCAAAGTCAAAG	
KAT8-siRNA-790	CCUCCAGGCAAAGAGAUUUTT	siRNA

The antibodies used (including the place of production and the dilution ratio) are as follows: Rabbit polyclonal anti-*Ci*IFN 1 (1:1000) and anti-*Ci*GAPDH (1:10000) antibodies were produced by our laboratory previously (36). Phospho-IRF3 (Ser386) rabbit monoclonal antibody (1:1000) and Phospho-TBK1/NAK (Ser172) (D52C2) XP^®^ Rabbit mAb (1:1000) were purchased from Beyotime (China) and CST (USA), respectively. Due to the high conservation between grass carp and human KAT8 (86%), anti-human KAT8 rabbit monoclonal antibody (HUABIO, China) was used to detect grass carp KAT8. Anti-IRF7 Ab (1:1000) was from HUABIO Biological and used as previously described (37). GFP-Tag and Flag-Tag Ab (1:5000) were purchased from Sigma (USA) and Abmart (USA), respectively. Acetylated-Lysine Antibody (1:1000) used to detect IRF3 and IRF7 acetylation level was purchased from Cell Signaling Technology (USA). The goat anti-mouse and anti-rabbit antibodies (1:10000) were from Sangon Biotech (Shanghai, China).

### Plasmids Construction and Transfection

Expression vectors encoding FLAG-tagged KAT8 were constructed by PCR cloning into pCMV-FLAG (Invitrogen, USA) eukaryotic expression vector. The ORF frame of *Ci*KAT8, *Ci*IRF3 and *Ci*IRF7 were separately inserted into pEGFP-C1 (Promega, USA) vector for co-IP, subcellular localization and DNA pull-down assays. The amino acid truncations KAT8-(△1-151), KAT8-(△151-264) and KAT8-(△264-487) were generated by PCR-based amplification and constructed into the pCMV-FLAG, pEGFP-C1 vector respectively. The ORF of *Ci*KAT8, *Ci*IRF3 and *Ci*IRF7 were separately inserted into pcDNA3.1 (+) vector (Invitrogen, USA) for over-expression experiments. IFN 1 and ISG15 promoter luciferase reporter plasmids were prepared in our lab (37); pISRE-TA-luc (beyotime, China) was used in luciferase assays. Each construction was confirmed by DNA sequencing. The primers for plasmid construction were given in **Table 1**.

For transient transfection of poly(I:C), B-DNA or Z-DNA into CIK cells, Lipofectamine 2000 (Invitrogen, USA) was used according to the manufacturer’s instructions. Empty plasmid or various expression plasmids were transfected in CIK cells and CO cells using Lipo8000^™^ transfection reagent (beyotime, China) according to the manufacturer’s protocol.

### The Expression Profile of KAT8 and Quantitative Real-Time PCR (qRT-PCR)

CIK cells transfected with 2 µg oligomeric nucleic acids [poly(I:C) or B-DNA or Z-DNA] or 50 µl of 10^−8^ TCID_50_ viruses (GCRV or SVCV) were separately cultured in 6-well plates for 6, 12, 24, 48, and 72 h, as described in our previous studies (37–39).

Total RNA was isolated from cells using RNAsimple Total RNA Kit (TIANGEN, China) following the instruction. cDNA was then synthesized from 1 µg RNA using PrimeScript RT Reagent Kit (TaKaRa, Japan). The qRT-PCR reactions were as follows: 7.2 µl of ddH2O, 10 µl of TB Green premix Ex Taq (TaKaRa, Japan), and 0.4 µl upstream and downstream primers for each. The cycling systems were as follows: 1 cycle of 5 min at 95°C, followed by 40 cycles of 30 s at 94°C, 30 s at 55°C, and 30 s at 72°C. The mRNA levels of KAT8, IFN 1 and ISG15 were detected by CFX Connect™ Real-Time System (Bio-Rad, USA). The expression value of each gene was normalized to *β-actin*, the results relative mRNA abundance was calculated by 2^-∆∆CT^ method. Gene-specific primer sequences for qRT-PCR were shown in **Table 1**.

### siRNA and Dual Luciferase Reporter Assays

In RNAi-mediated gene knockdown assay, the siRNAs against *Ci*KAT8 and negative control RNA (N.C) oligonucleotides were synthesized by Suzhou GenePharma Co., Ltd (**Table 1**). The instruction of transfection of siRNA has been reported in the previous study (40).

In dual luciferase reporter assays, CO cells were seeded on 24-well dishes (NEST Biotechnology, China) and transfected with the indicated plasmids. At 24 h post transfection, cells protein was extracted and luciferase activity was measured by the dual Luciferase reporter gene assay kit (Promega, USA). The plasmid of PRL-TK was served as the reference in the assay.

### Subcellular Localization Analysis

Nuclear localization of KAT8 protein in grass carp was predicted by cNLS Mapper software (http://nls-mapper.iab.keio.ac.jp/cgi-bin/NLS_Mapper_form.cgi). CIK cells, plated on microscopic cover glass (NEST Biotechnology, China), were transfected with the following plasmids (pEGFP-KAT8, pEGFP-KAT8-(△1-151), pEGFP-KAT8-(△151-264), pEGFP-KAT8-(△264-487), respectively. After 24 h of transfection, the cells were fixed in 4% (wt/vol) paraformaldehyde for 20 min and then stained with 0.1 µg/ml DAPI (Sigma-Aldrich) at 37˚C for 15 min. Cells were observed with a confocal laser microscope (FV1000; Olympus).

### Immunoblot (Western Blot) Analysis, Coimmunoprecipitation (CO-IP) Assays and Analysis of Acetylation State

CO cell protein extracts were prepared by standard techniques and subjected to Western blot to detect the protein level of IFN 1, KAT8 and GAPDH. The methods were described as our previous studies (37, 41).

In CO-IP experiments, CO cells seeded in 60 mm dishes were transfected with a total of 3 µg indicated plasmids. 36 h post-transfection, cells protein was harvested in NP40 lysis buffer and incubated with FLAG/GFP Ab tagged agarose (Sigma, USA) or IgG tagged agarose (Sigma, USA). The immunoblotting method was used to detect the interaction according to the manufacturer’s protocol, and the detailed experimental steps were performed as described previously (36).

In the absence of the specific anti-acetyl-IRF3 and anti-acetyl-IRF7 antibodies, immunoprecipitation assays were performed to study IRF3 and IRF7 acetylation. CO cells seeded in 60 mm dishes were transfected with Flag-tagged KAT8-WT or mutant KAT8 plus GFP-tagged IRF3/GFP-tagged IRF7. At 36 h post-transfection, the medium was removed and washed twice with PBS. The cells were lysed on ice with NP40 lysing buffer containing deacetylase inhibitor cocktail (1:100) (Beyotime, China) for 30min. Then they were centrifuged at 12000rpm for 10min, the supernatant was collected and incubated with GFP Ab tagged agarose (KT-HEALTH, China) overnight at 4°C. The beads were washed with PBS three times and eluted with 100 µl 2 × SDS loading buffer by boiling for 10 min at 95°C. The precipitates were detected by immunoblotting with the acetylated-lysine antibody.

### DNA Pull-Down Assays

DNA pull-down assays were carried out as described previously (38, 42). In brief, CO cells were transfected with the indicated plasmids (CMV-Flag+IRF3/IRF7-GFP, KAT8-Flag+IRF3/IRF7-GFP) for 36h, and cells protein was extracted for further experiments. Meanwhile, biotin-labeled interferon-sensitive response element (ISRE) and non-biotin-labeled ISRE (Sangon Biotech, China) (**Table 1**) were incubated with M-280 Streptavidin-Coupled Dynabeads for 15 min. Then the extracted cells protein was incubated with the prepared M-280 Streptavidin-Coupled Dynabeads at 4°C for 12 h, and the magnetic beads were treated with 2 × SDS sample buffer at 95 °C for 10 min. Western blot was used to detect the combination of IRF3/IRF7 with ISRE response element.

### Antiviral Activity Test

To investigate the antiviral activity of *Ci*KAT8, CIK cells were seeded in 6-well plates and transfected with 2 μg of pcDNA3.1-KAT8 for 24 h. Then cells were infected with 50 µl 10^-8^ TCID_50_ GCRV-873 for 5 day. Among them, MOCK group was the no treatment group. Then the cells were fixed with 4% paraformaldehyde for 30 min, and stained with 1% (w/v) crystal violet for 30 min for visualizing cytopathic effect (CPE). The three viral genes of GCRV-873, vp5 (Genbank ID: AF403391.1), vp6 (Genbank ID: HQ018818.1) and vp7 (Genbank ID: HQ018819.1), were detected by qRT-PCR. Gene-specific primer sequences for qRT-PCR were shown in **Table 1**.

### Statistical Analysis

Each data of qRT-PCR and dual-luciferase assays were presented as mean and ± SD (n = 3). Statistical significance was determined with the two-tailed Student’s t-test, with a *p* value of less than 0.05 considered statistically significant. Each figure of western blot, cytopathic effect assay and confocal microscopy was produced using Image J, which was represented by three independent experiments in this study.

## Results

### *Ci*KAT8 Is Induced by the Exogenous Nucleic Acids and Viruses

**Figure 1:**
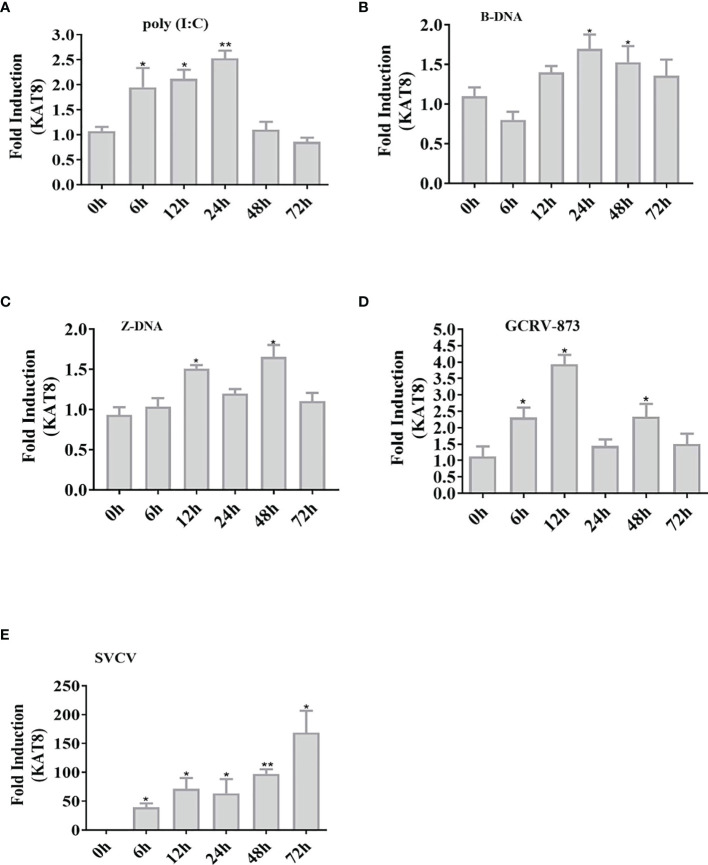
Nucleic acids increase the expression of *CiKAT8.* CIK cells seeded in 6-well plates were transfected with (poly I:C, B-DNA, and Z-DNA) **(A–C)** or infected with viruses (GCRV-873, SVCV) **(D, E)** for different time (0 h, 6 h, 12 h, 24 h, 48 h, and 72 h). qRT-PCR analyzed the mRNA level of KAT8 in CIK cells. *β-actin* was served as an internal reference. The group of 0 h was a control. * represented significant (*p* < 0.05) and ** represented highly significant (*p* < 0.01).

The expression pattern of *CiKAT8* was significantly up-regulated in cells transfected with 2 µg poly I:C, B-DNA and Z-DNA respectively (**Figures 1A–C**). In addition, the expression of *CiKAT8* was also significantly up-regulated after GCRV and SVCV infection (**Figures 1D, E**). These data demonstrated that DNA, RNA analogues (poly I:C) and viruses can up-regulate KAT8 expression.

### *Ci*KAT8 Suppresses IFN 1 and ISG15 Expression

**Figure 2:**
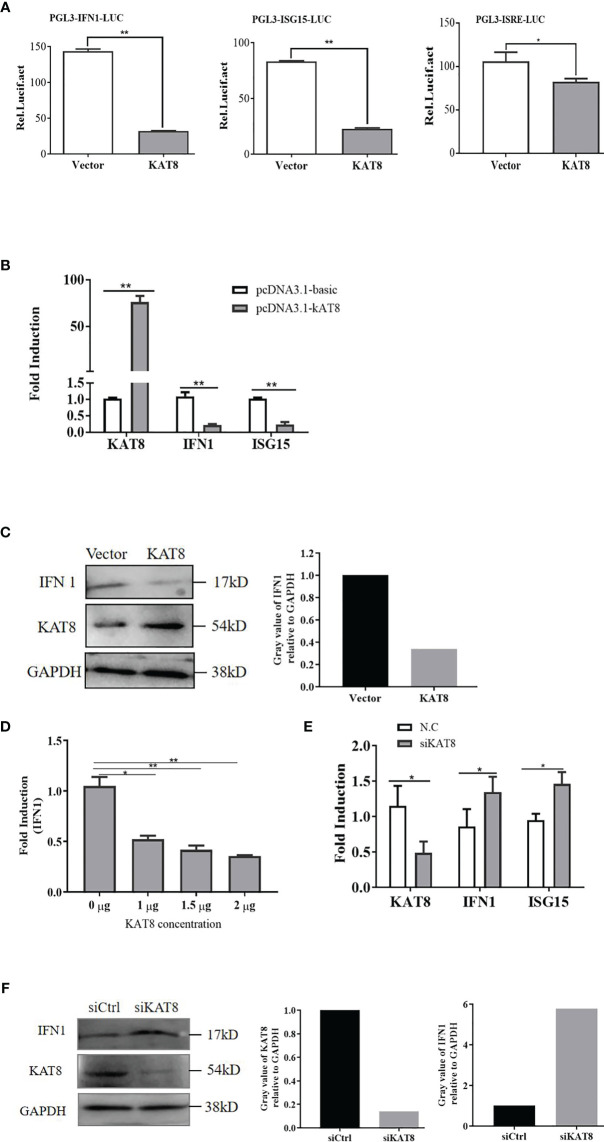
*Ci*KAT8 suppresses the production of IFN 1. CO cells were transfected with 2 µg of pcDNA3.1-KAT8 or empty vector (pcDNA3.1-basic). **(A)** Dual-luciferase analyzed the activities of IFN 1, ISG15 promoters and ISRE element. **(B)** qRT-PCR was used to detect IFN 1 and ISG15 expression. **(C)** Western bolt analyzed the expression of IFN 1 and KAT8 in CO cells. GAPDH served as an internal reference. **(D)** CO cells were transfected with pcDNA3.1-KAT8 at three different dosages (1, 1.5, or 2 µg), qRT-PCR analyzed the mRNA level of IFN 1. **(E, F)** CIK cells were transfected with control siRNA (N.C/siCtrl) or specific siRNA targeting KAT8 (siKAT8) for 36 h, and the levels of IFN 1, ISG15 mRNA and IFN 1 protein were analyzed by qRT-PCR and Western blot, respectively. * (*p* < 0.05), ** (*p* < 0.01). Data are representative of three independent experiments **(C, F)**. The histograms exhibit the relative expression levels, which are quantified using ImageJ software.

To confirm the potential role of *Ci*KAT8 in innate immune, IFN 1 and ISG15 expression were detected in cells transfected with *Ci*KAT8. In reporter assays, overexpression of *Ci*KAT8 significantly inhibited *IFN 1*, *ISG15* promoters and IFN-stimulated response element (ISRE) promoter activity in CO cells (**Figure 2A**). The mRNA levels of these genes in CO cells overexpressing KAT8 were markedly reduced (**Figure 2B**). In addition, we observed that the protein level of IFN 1 was also significantly inhibited when CO cells were overexpressed with KAT8 (**Figure 2C**). Further experiments indicated that the overexpression of KAT8 inhibited the transcription of IFN 1 in a dose-dependent manner (**Figure 2D**). On the contrary, knockdown of KAT8 in CIK cells substantially enhanced the expression of IFN 1 (both at mRNA and protein level) and ISG15 (**Figures 2E, F**). Collectively, these data demonstrated that KAT8 inhibited the production of IFN 1 and ISG15 in CO and CIK cells and negatively regulates innate immune response.

### *Ci*KAT8 Negatively Regulates Cellular Antiviral Response

**Figure 3:**
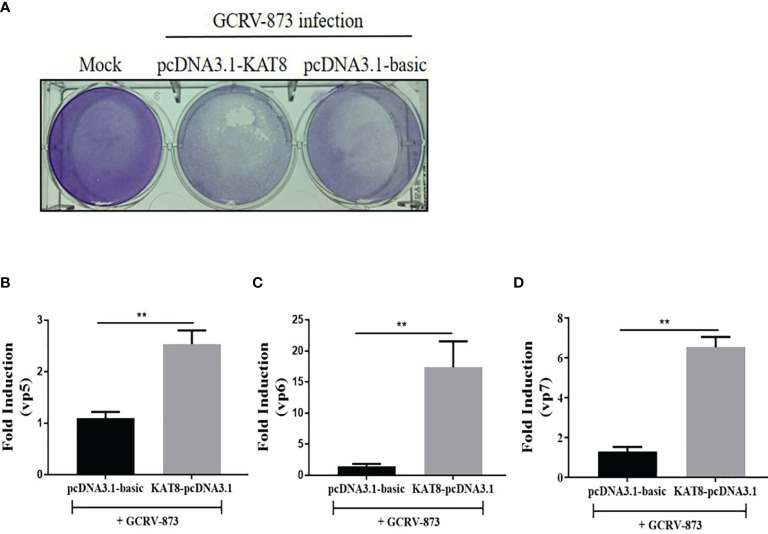
*Ci*KAT8 enhances virus replication in GCRV873-infected CIK cells. CIK cells were transfected with 2 μg of pcDNA3.1-KAT8 for 24 h. Then cells were infected with 50 µl 10^-8^ TCID_50_ GCRV-873 for 5 day. The cells were fixed with 4% paraformaldehyde and stained with 1% crystal violet **(A)**. **(B–D)** CIK cells were transfected with 2 μg of pcDNA3.1-KAT8 and then infected with GCRV-873 (10^-8^ TCID_50_). After 5 day, total RNA were extracted for detecting the mRNA levels of the vp5, vp6, and vp7 of GCRV-873 by qRT-PCR analysis ** (*p* < 0.01).

To further evaluate the antiviral effect of grass carp KAT8, we performed cytopathic effect (CPE) assays. Overexpression of KAT8 in CIK cells caused CPE to enhance upon challenged with GCRV-873 (10^-8^ TCID_50_) (**Figure 3A**). As expected, some GCRV-873 replication related genes, including VP5 gene, VP6 gene and VP7 gene, were significantly increased in CIK cells with overexpressed KAT8 (**Figures 3B–D**).

### *Ci*KAT8 Inhibits IFN 1 Expression *Via* IRF3/IRF7 Signaling Pathway

**Figure 4:**
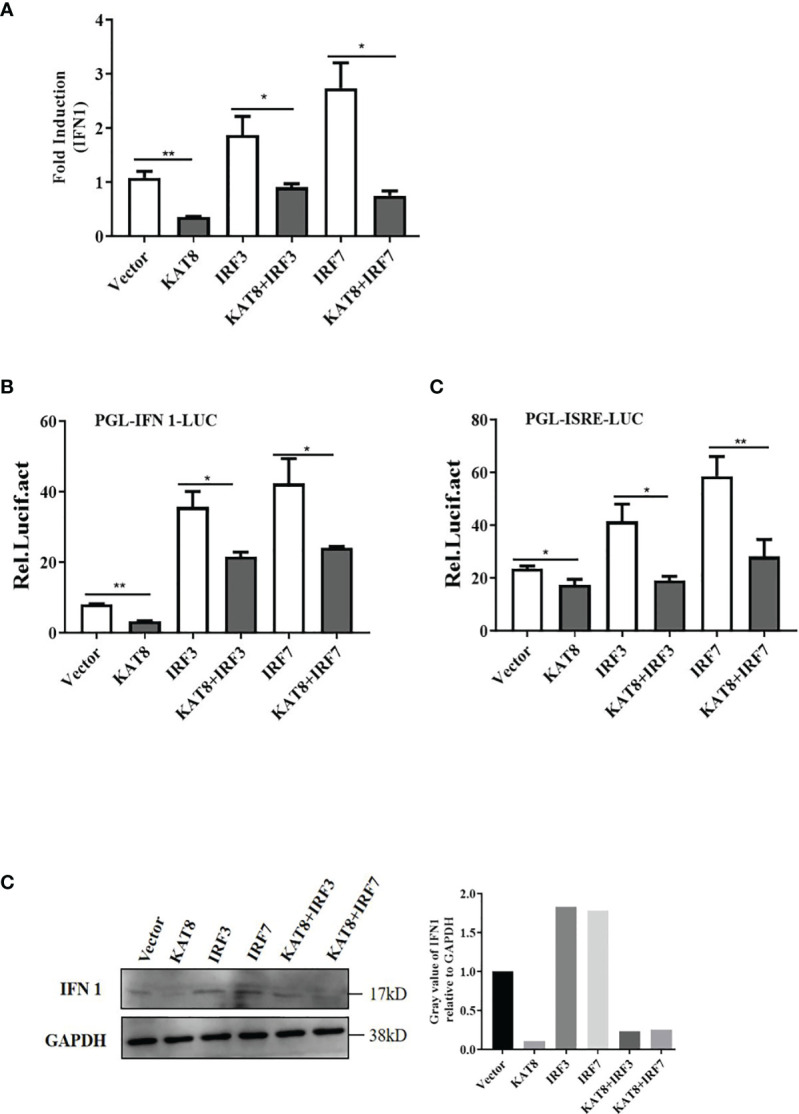
*Ci*KAT8 blocks IRF3/IRF7-induced IFN 1 expression. CO cells seeded in (6-well or 24-well) plates were single-transfected or co-transfected with 2 µg of pcDNA3.1-KAT8 and pcDNA3.1-IRF3/IRF7. pcDNA3.1-basic was used as a control group. 36 h later, cells were harvested for detection of the mRNA level of IFN 1 **(A)** or luciferase activities of IFN 1 promoter and ISRE element **(B)**, or the protein level of IFN 1 **(C)**. * (*p* < 0.05) and ** (*p* < 0.01). Data are representative of three independent experiments **(C)**. The histograms exhibit the relative expression levels, which are quantified using ImageJ software.

IRF3 and IRF7 are the main transcription factors involved in IFN 1 production. To investigate the relationship between KAT8 and IRF3/IRF7, several set of experiments were performed. CO cells were divided into single transfection group (KAT8-pcDNA3.1, IRF3-pcDNA3.1 or IRF7-pcDNA3.1) and co-transfection group (KAT8-pcDNA3.1 + IRF3-pcDNA3.1, KAT8-pcDNA3.1 + IRF7-pcDNA3.1). In a single transfection group, overexpression of grass carp IRF3 or IRF7 led to a significant activation of IFN 1 response, which is opposite to the cells with KAT8-pcDNA3.1. However, the IFN 1 response was weakened when cells were co-transfected with pcDNA3.1-KAT8 and pcDNA3.1-IRF3 or pcDNA3.1-IRF7 (**Figures 4A–C**). Furthermore, it is necessary to investigate whether KAT8 affected the phosphorylation of the proteins in type I IFN signaling pathway. The results showed that there were no detectable difference of IRF3, IRF7 and TBK1 phosphorylation levels (**Figure S1**). These results indicated that KAT8 down-regulated IFN 1 response not by affecting the phosphorylation level of IRF3, IRF7 and TBK1.

### KAT8 Protein Locates in the Nucleus

**Figure 5:**
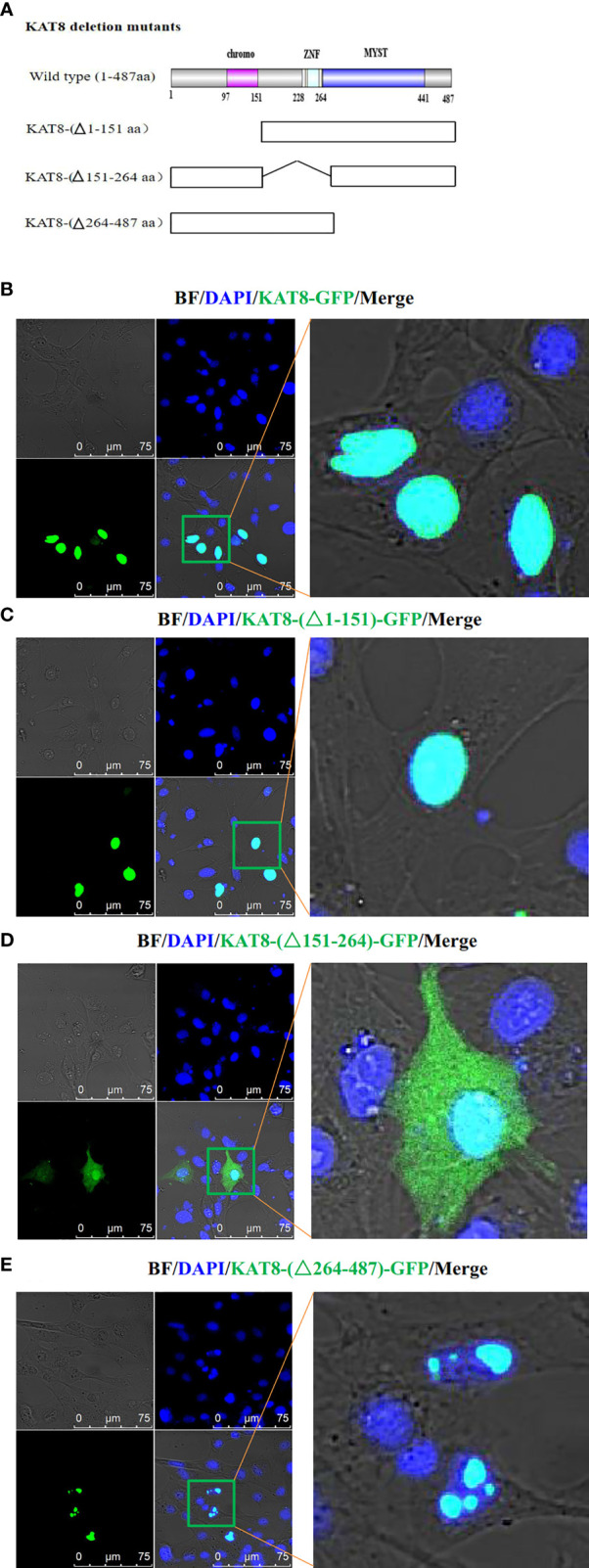
Subcellular localization of *Ci*KAT8. **(A)** Structural diagram of wild-type and mutants KAT8. **(B–E)** CIK cells seeded on microscopy dishes were separately transfected with 2 µg of KAT8-GFP or KAT8-(△1-151)-GFP or KAT8-(△151-264)-GFP or KAT8-(△264-487)-GFP. 24 hours later, the cells were fixed and examined using a confocal microscopy (gray, Brightfield/BF; green, pEGFP-KAT8 or mutants pEGFP-KAT8; blue, DAPI). Scale bar is 75 µm.

To further explore the physiological functions of CiKAT8, the nuclear localization signal of KAT8 protein was predicted by cNLS Mapper software. The result showed that the NLS sequence of KAT8 is located in 169-178 aa. Then subcellular localization of KAT8 was performed in CIK cells. We constructed three different GFP-tagged KAT8 truncations, respectively lacking the chromatin domain (△1-151), C2HC zinc fingers (△151-264) and the enzymatic MYST domain (△264-487) (**Figure 5A**). After KAT8 and the mutants plasmids were separately transfected into CIK cells, we observed that KAT8-WT and KAT8-(△1-151) were evenly distributed throughout the nucleus (**Figures 5B, C**). The mutant KAT8-(△151-264) without NLS sequence was disorderly distributed both in the nuclear and cytoplasm (**Figure 5D**). Surprisingly, the mutant KAT8-(△264-487) was located in the nucleus in the form of aggregates (**Figure 5E**). These results showed that KAT8 and the truncated KAT8 mutants had different localization, suggesting their different functions in immune signaling pathways.

### *Ci*KAT8 Directly Interacts With IRF3/IRF7 *Via* Its MYST Domain

**Figure 6:**
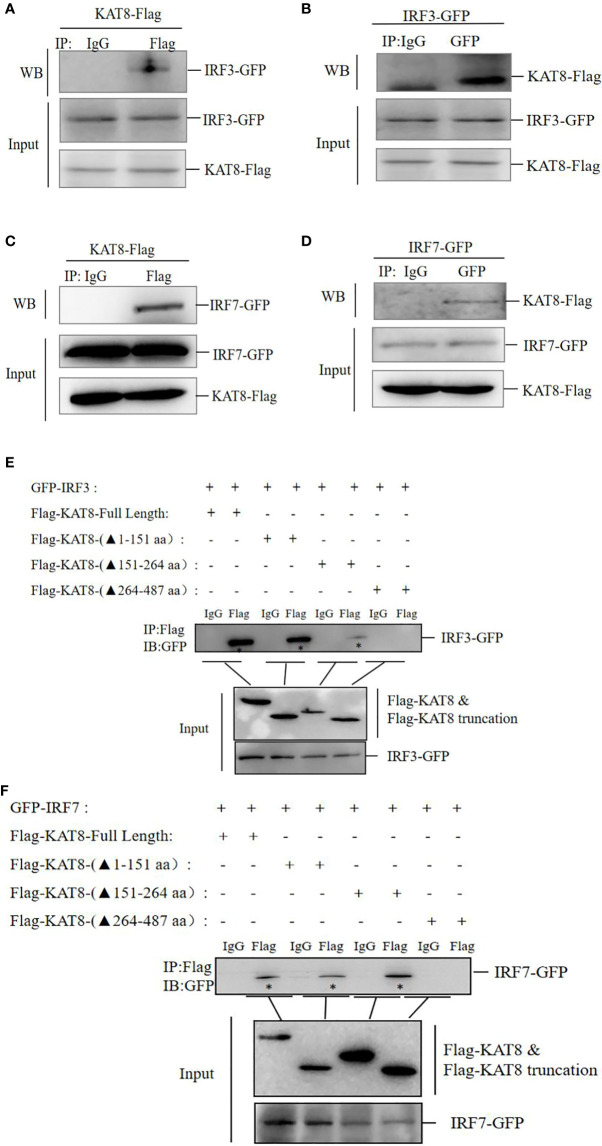
*Ci*KAT8 interacts with IRF3 and IRF7 *via* the MYST domain. **(A–D)** CO cells seeded in 60mm dishes were co-transfected with 1.5 µg of KAT8-Flag and 1.5 µg of (IRF3-GFP or IRF7-GFP) plasmids, 36 h later, cell lysates were immunoprecipitated with anti-Flag Ab **(A, C)** or anti-GFP Ab **(B, D)**, then Western blot analyzed the interaction of KAT8 with IRF3/7. **(E, F)** CO cells seeded in 60 mm dishes were transfected with 1.5 µg KAT8-Flag or Flag-tagged mutant KAT8 (△1-151, △151-264, △264-487) plus (IRF3-GFP or IRF7-GFP), 36 h later, cell lysates were immunoprecipitated with anti-Flag Ab, Western blot analyzed the interaction of three KAT8 truncations with IRF3/IRF7. “*” refers to IRF3-GFP or IRF7-GFP. The molecular masses of KAT8-Flag, KAT8-(△1-151)-Flag, KAT8-(△151-264)-Flag, KAT8-(△264-487)-Flag, IRF3-GFP, IRF7-GFP are 60, 42, 46, 34, 85, and 82 kD, respectively. Data are representative of three independent experiments.

Co-IP assay showed that FLAG-tagged KAT8 separately interacted with GFP-tagged IRF3 and GFP-tagged IRF7 in CO cells (**Figures 6A–D**). To map KAT8 domains required for the interaction with IRF3 and IRF7. Among KAT8 truncations, only those containing MYST domain (△1-151 and △151-264) were able to interact with IRF3 and IRF7, that lacking MYST domain (△264-487) could not (**Figures 6E, F**).

### *Ci*KAT8 Promotes the Acetylation of IRF3 and IRF7

**Figure 7:**
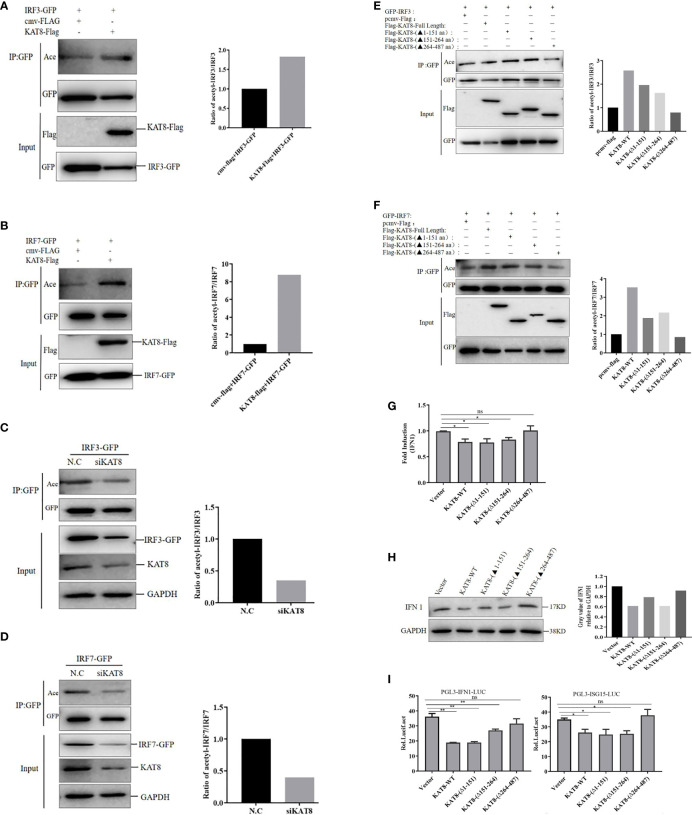
*Ci*KAT8 acetylates IRF3/7 *via* its MYST domain. **(A, B)** CO cells seeded in 60 mm dishes were co-transfected with 1.5 µg of cmv-Flag or KAT8-Flag and 1.5 µg of IRF3-GFP plasmids **(A)**, or 1.5 µg of cmv-Flag or KAT8-Flag and IRF7-GFP plasmids **(B)**. 36 h later, the cell lysates were added with the deacetylase inhibitors cocktail and then co-immunoprecipitated with an anti-GFP antibody. Eventually, Western blot was performed with the indicated antibodies. **(C, D)** CO cells were transfected with control siRNA (N.C) or specific siRNA targeting KAT8 (siKAT8) for 12 h, then transfected with IRF3-GFP **(C)** or IRF7-GFP **(D)** plasmids for other 24 h, respectively. The subsequent experiment was conducted similarly to **(A, B, E, F)** Effect of KAT8 and its truncated mutants on the acetylation of IRF3/IRF7, CO cells were transfected with the indicated plasmids for 36 h, and the subsequent experiment was conducted similarly to A, **(B)** The molecular masses of KAT8-Flag, KAT8-(△1-151)-Flag, KAT8-(△151-264)-Flag, KAT8-(△264-487)-Flag, IRF3-GFP, IRF7-GFP are 60, 42, 46, 34, 85, and 82 kD, respectively. **(G–I)** CO cells seeded in plates (6-well or 24-well) were transfected with 2 µg of pcDNA3.1-KAT8-WT or pcDNA3.1-KAT8-(△1-151) or pcDNA3.1-KAT8-(△151-264) or pcDNA3.1-KAT8-(△264-487). 36 h later, cells were harvested for detection of the mRNA level of IFN 1 **(G)** or the protein level of IFN 1 **(H)** or the luciferase activities of IFN 1 and ISG15 promoters **(I)** * (p < 0.05) and ** (p < 0.01). Data are representative of three independent experiments **(A–F, H)**. The histograms show relative expression levels quantified using the Image J software. "ns" means no significance.

The above conclusions have proved that KAT8 interacted with IRF3 and IRF7 through MYST domain (acetyltransferase domain), so whether KAT8 regulates IFN 1 expression by affecting the acetylation level of IRF3 and IRF7. We then analyzed the acetylation of IRF3/IRF7 in CO cells co-transfected Flag-tagged KAT8 (WT) with GFP-tagged IRF3 or GFP-tagged IRF7. We used acetylated-Lysine antibody to detect the acetylation of IRF3 and IRF7 and found that KAT8 enhanced the acetylation level of IRF3 and IRF7 (**Figures 7A, B**). In contrast, the acetylation level of IRF3 and IRF7 were suppressed by knockdown of KAT8 (**Figures 7C, D**).

Furthermore, we then analyzed the acetylation of IRF3 and IRF7 in CO cells transfected with Flag-tagged KAT8 (WT) and the three different Flag-tagged KAT8 truncations. Each of KAT8-WT, KAT8-(△1-151) and KAT8-(△151-264) can enhance IRF3/IRF7 acetylation. This effect disappeared when the MYST domain of KAT8 (KAT8-(△264-487) was deleted (**Figures 7E, F**). Overall, these data indicated that KAT8 can enhance the acetylation of IRF3 and IRF7 through its MYST domain.

Then, to further investigate the necessity of MYST domain of KAT8 in regulating IFN 1 expression, KAT8 and three truncations of KAT8 were overexpressed in CO cells, respectively. The mRNA and protein expression of IFN 1 were detected by qRT-PCR and Western blot. Without the MYST domain of KAT8 (KAT8-△264-487) did not affect IFN 1 expression (both at mRNA and protein levels) (**Figures 7G, H**). Consistent with this conclusion, in reporter assays, KAT8 lacking the MYST domain cannot inhibit IFN 1 and ISG15 promoter activities (**Figure 7I**).

### *Ci*KAT8 Impairs the Combination of IRF3/IRF7 to ISRE Response Element *In Vitro*

**Figure 8:**
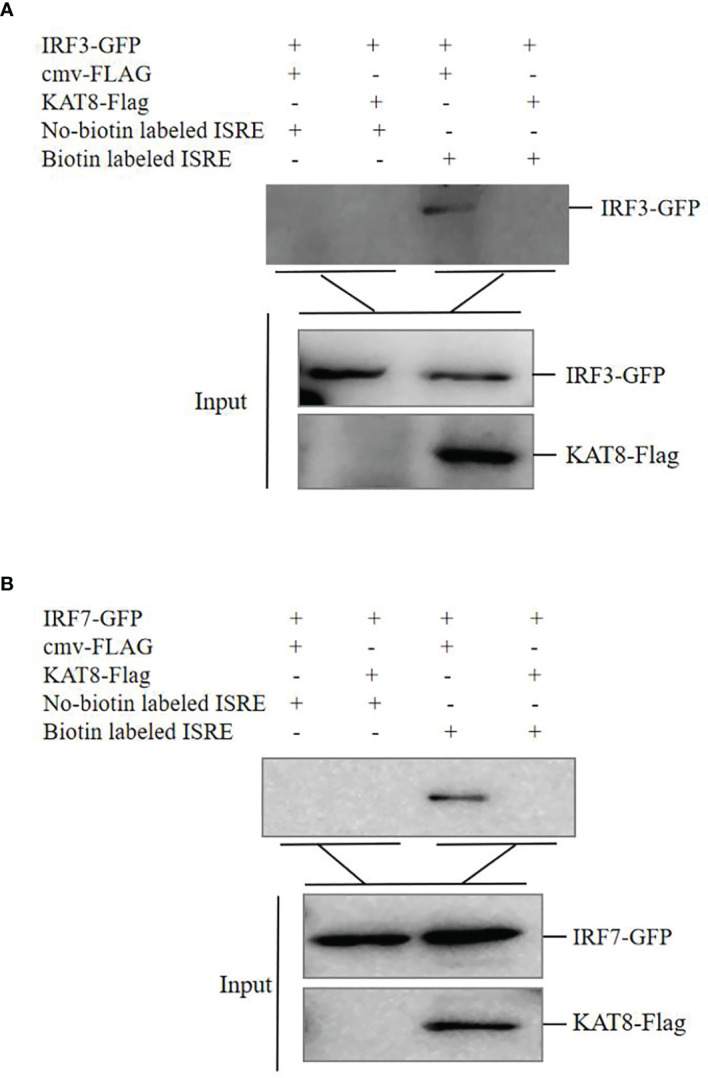
*Ci*KAT8 inhibits the combination of IRF3/IRF7 with ISRE response element. **(A, B)** CO cells seeded in 60 mm dishes and transfected with 1.5 µg of cmv-FLAG or KAT8-FLAG puls 1.5 µg of IRF3-GFP or IRF7-GFP. 36 h later, the cell extracts were incubated with immobilized biotin-labeled ISRE or No-biotin labeled ISRE. 12 h later, the beads were washed with lysis buffer three times, eluted with 2 × SDS sample buffer and boiled at 95°C for 10 min. Western blot was used to detect the affinity of IRF3/IRF7 with ISRE response element. Data are representative of three independent experiments.

To further reveal molecular mechanism of KAT8 in negatively regulating the activity of IRF3 and IRF7, DNA-pulldown assay was performed. Overexpression of KAT8 in CO cells inhibited the binding of IRF3/IRF7 to ISRE response element (**Figures 8A, B**). Collectively, these may suggest that the acetylation of IRF3 and IRF7 by KAT8 will promote IRF3 and IRF7 break away from ISRE element and reduces their activities.

## Discussion

In mammals, KAT8 is the major enzyme that catalyzes the acetylation of H4K16 (26). The recent study showed that KAT8 is universally expressed in a variety of immune cells (35). In this study, grass carp KAT8 was up-regulated under poly I:C, B-DNA and Z-DNA stimulation or viruses (GCRV-873 and SVCV) infection (**Figure 1**). These may indicate that fish KAT8 is also involved in the innate immune response. Therefore, it is urgent to uncover the role of fish KAT8 in innate immune response.

In mice, KAT8 selectively restrains RNA and DNA virus-induced IFN-I (35). Similarly, we also found grass carp KAT8 can inhibit the expression of IFN 1 and ISG15. So it negatively regulate the cellular antiviral response (**Figure 2**, **3**). Subsequently, we found that grass carp KAT8 specifically inhibited IRF3/IRF7-induced innate immune response (**Figures 4A–C**). Therefore, the interaction mode of IRF3/IRF7 and KAT8 needs to be confirmed. IRF3 and IRF7 are the main transcription factors involved in IFN 1 production (43, 44). It is known that phosphorylation is linked to the activated formation of IRF3 and IRF7 (45–47). However, grass carp KAT8 did not affect the phosphorylation level of IRF3 and IRF7 (**Figure S1**). Therefore, KAT8 controlled IRF3 and IRF7 in other ways.

Currently, it has been report that there are many types of protein post-translational modifications, including phosphorylation, ubiquitination, SUMOylation, methylation, acetylation and so on. There may have several kinds of PTMs for some specific proteins, such as IRF3 and IRF7, to regulate their activities. For instance, GPATCH3, PKZ and NEK6 regulate the phosphorylation and nuclear translocalization of IRF3/IRF7 to control IFN 1 response (37, 39, 41); VP35 represses IFN transcription by increasing PIAS1-mediated SUMOylation of IRF7 (48); Methyltransferase NSD3 catalyzes the methylation of IRF3 at K366 to enhance antiviral immune response (49); Recently, acetylation also has been reported as an unconventional modification to regulate the activities of IRF3 and IRF7 (50, 51). The various and complicated PTMs may effectively regulate immune homeostasis in cells. Therefore, exploring the effect of fish KAT8 on IRF3/IRF7 is meaningful to expand the knowledge of PTMs.

KAT8, an acetyltransferase, participates in a variety of cellular physiological processes by mediating the acetylation of proteins (29–31). Human KAT8 is identified as a histone acetyltransferase in the nucleus (28, 52). The subcellular localization assays also showed grass carp KAT8 mainly locates in the nucleus to perform its function. However, the mutant (deletion of MYST domain), has the abnormal localization, which results in the loss of the relevant functions (**Figure 5**). MYST domain is critical for acetylation activity of acetyltransferase (27–29). Grass carp KAT8 directly interacted with IRF3 and IRF7 and then promoted their acetylation through its MYST domain (**Figures 6**, **7**). Therefore, controlling the acetylation of IRF3/IRF7 is the ‘right way’ of KAT8 for inhibiting IFN I response.

It is known that the acetylation of IRF3 and IRF7 controls innate immune response. Acetylation modification may induce the changes of protein conformation, thereby directly affecting protein-DNA interactions (21). Brd3 controls the acetylation of IRF3 to affect the affinity of IRF3-p300 complex with IFNb1 promoter (50). IRF7 is acetylated by p300/CBP-associated factor (PCFA) and GCN5, which impairs the affinity of IRF7 with DNA (51). Similarly, the extracellular experiment showed that the acetylation of IRF3 and IRF7 by KAT8 promotes them to break away from ISRE response element (**Figure 8**).

In conclusion, our findings provide a new theoretical basis for the negative effects of fish KAT8 in the innate immune signaling pathways. We identified fish KAT8 as a negative regulator that reduces IFN 1 expression by directly binding and acetylating the transcription factors IRF3/IRF7, thereby preventing the combination of IRF3/IRF7 with ISRE response element. The current finding helps us understand how innate antiviral response is delicately modulated to achieve homeostasis.

## Data Availability Statement

The datasets presented in this study can be found in online repositories. The names of the repository/repositories and accession number(s) can be found in the article/**Supplementary Material**.

## Author Contributions

CH and XX supervised the research. DL revised the manuscript. ML conceived the study, designed and performed the experiments. ML, XX, HM and JH analyzed the experiments and data. ZJ, ZS, and TY provided reagents, technical assistance and contributed to completion of the study. ML wrote the manuscript. All authors reviewed the results and approved the final version of the manuscript.

## Funding

This work supported by research grants from the National Natural Science Foundation of China (32160871, 31960735 and 32160872), Jiangxi Agriculture Research System (JXARS-06), China Postdoctoral Science Foundation (2019M662279), Jiangxi Postdoctoral Science Foundation (2019KY43, 2020RC22).

## Conflict of Interest

The authors declare that the research was conducted in the absence of any commercial or financial relationships that could be construed as a potential conflict of interest.

## Publisher’s Note

All claims expressed in this article are solely those of the authors and do not necessarily represent those of their affiliated organizations, or those of the publisher, the editors and the reviewers. Any product that may be evaluated in this article, or claim that may be made by its manufacturer, is not guaranteed or endorsed by the publisher.
