# Supplementary material for: Grass Carp (Ctenopharyngodon idella) KAT8 Inhibits IFN 1 Response Through Acetylating IRF3/IRF7
Source: Front Immunol. 2022 Jan 3;12:808159. doi: 10.3389/fimmu.2021.808159 (PMC8761793; doi:10.3389/fimmu.2021.808159)
Supplement: Supplementary file 1 [file DataSheet_1.doc]

Fig. S1

1. (B)


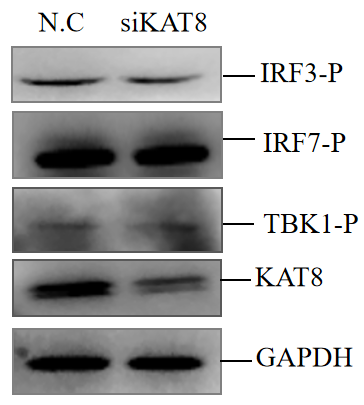

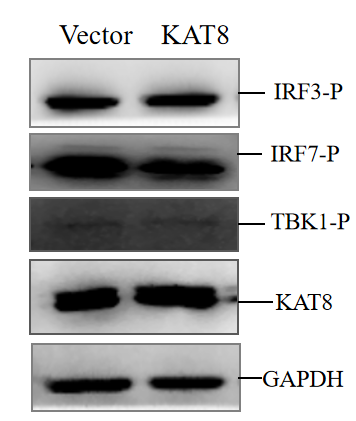


**Fig. S1. *Ci*KAT8 does not affect the phosphorylation of IRF3, IRF7, TBK1**

(A) CO cells were seed into 6-well plates, and then transfected with 2 µg of pcDNA3.1-basic (Vector) or 2 µg of pcDNA3.1-KAT8. 36 h later, total cell protein was extracted and the phosphorylation of IRF3, IRF7 and TBK1 was analyzed by Western blot. (B) CIK cells were transfected with control siRNA (N.C) or specific siRNA targeting KAT8 (siKAT8) for 36 h, whole-cell lysates were prepared for Western blot analysis. “p” in the front of IRF3, IRF7 and TBK1 indicates their phosphorylation.
